# Supplementary material for: End Sequence Analysis Toolkit (ESAT) expands the extractable information from single-cell RNA-seq data
Source: Genome Res. 2016 Oct;26(10):1397–410. doi: 10.1101/gr.207902.116 (PMC5052061; doi:10.1101/gr.207902.116)
Supplement: Supplemental Material [file supp_gr.207902.116_Supp_Materials.tar.gz › ESAT-esat_v0.1/doc/html/namespaces.html]

ESAT: Packages


ESAT


- Main Page
- Related Pages
- Packages
- Classes
- Files

- Packages


All Classes Namespaces Files Functions Variables Enumerator Pages

Packages

Here are the packages with brief descriptions (if available):

[detail level 12345]

|  |  |
| --- | --- |
| ▼Nbroad |  |
| ▼Ncore |  |
| Nannotation |  |
| Ndatastructures |  |
| Nerror |  |
| Nhmm |  |
| Nmath |  |
| ▼Nmotif |  |
| Nmeme |  |
| Nmisc |  |
| Nmultiplealignment |  |
| Noverlaputils |  |
| Nparser |  |
| Nprimer3 |  |
| Nsequence |  |
| ▼Nsiphy |  |
| ▼Ntools |  |
| Nconservation |  |
| Nutil |  |
| ▼Npda |  |
| Nannotation |  |
| Ncountreads |  |
| Ndatastructures |  |
| NdifferentialExpression |  |
| Nenrichment |  |
| ▼Nfeature |  |
| Ngenome |  |
| Ngene |  |
| ▼Ngeneexpression |  |
| ▼Nagilent |  |
| Nanalysis |  |
| Nclustering |  |
| ▼Nrap |  |
| Nmisc |  |
| Nrnai |  |
| Nsamtools |  |
| ▼Nseq |  |
| Nalignment |  |
| Nchip |  |
| Nclip |  |
| Nfastq |  |
| Ngraph |  |
| Nprotection |  |
| ▼Nrap |  |
| Nrna |  |
| ▼Nsegmentation |  |
| Nutils |  |
| Ntools |  |
| ▼Nprojection |  |
| Ngsa |  |
| ▼Njaligner |  |
| Nformats |  |
| Nmatrix |  |
| Nutil |  |
| ▼NJama |  |
| Nutil |  |
| ▼Nnextgen |  |
| ▼Ncore |  |
| NreadFilters |  |
| ▼Numms |  |
| ▼Ncore |  |
| Nalignment |  |
| ▼Nannotation |  |
| Nfilter |  |
| Ncoordinatesystem |  |
| Nexception |  |
| Nfeature |  |
| Ngeneral |  |
| ▼Nmodel |  |
| Nscore |  |
| Nreaders |  |
| NreadFilters |  |
| ▼Nscripture |  |
| Nstatistics |  |
| Nutils |  |
| Nwriters |  |
| Nesat |  |


---

Generated on Fri Aug 15 2014 15:11:16 for ESAT by  

 1.8.7
